# Effect of Na- and Organo-Modified Montmorillonite/Essential Oil Nanohybrids on the Kinetics of the In Situ Radical Polymerization of Styrene

**DOI:** 10.3390/nano11020474

**Published:** 2021-02-13

**Authors:** Ioannis S. Tsagkalias, Alexandra Loukidi, Stella Chatzimichailidou, Constantinos E. Salmas, Aris E. Giannakas, Dimitris S. Achilias

**Affiliations:** 1 Department of Chemistry, Aristotle University of Thessaloniki, 54124 Thessaloniki, Greece; itsagkal08@gmail.com (I.S.T.); loukidialexia@gmail.com (A.L.); stedimcha@chem.auth.gr (S.C.); 2 Department of Material Science & Engineering, University of Ioannina, 45110 Ioannina, Greece; ksalmas@uoi.gr; 3 Department of Food Science and Technology, University of Patras, 30100 Agrinio, Greece; agiannakas@upatras.gr

**Keywords:** polymerization kinetics, polystyrene, Na-montmorillonite, organo-modified montmorillonite, thyme oil, oregano oil, basil oil

## Abstract

The great concern about the use of hazardous additives in food packaging materials has shown the way to new bio-based materials, such as nanoclays incorporating bioactive essential oils (EO). One of the still unresolved issues is the proper incorporation of these materials into a polymeric matrix. The in situ polymerization seems to be a promising technique, not requiring high temperatures or toxic solvents. Therefore, in this study, the bulk radical polymerization of styrene was investigated in the presence of sodium montmorillonite (NaMMT) and organo-modified montmorillonite (orgMMT) including thyme (TO), oregano (OO), and basil (BO) essential oil. It was found that the hydroxyl groups present in the main ingredients of TO and OO may participate in side retardation reactions leading to lower polymerization rates (measured gravimetrically by the variation of monomer conversion with time) accompanied by higher polymer average molecular weight (measured via GPC). The use of BO did not seem to affect significantly the polymerization kinetics and polymer MWD. These results were verified from independent experiments using model compounds, thymol, carvacrol and estragol instead of the clays. Partially intercalated structures were revealed from XRD scans. The glass transition temperature (from DSC) and the thermal stability (from TGA) of the nanocomposites formed were slightly increased from 95 to 98 °C and from 435 to 445 °C, respectively. Finally, better dispersion was observed when orgMMT was added instead of NaMMT.

## 1. Introduction

In recent years, the great concern about the use of hazardous and/or toxic additives in food packaging materials has led the scientific community to search for new bio-based compounds to be used in active plastic packaging. Among natural antioxidants, essential oils are the most widely used materials in the food packaging industry [1]. To this direction, hybrids of montmorillonite (MMT) with essential oils (EO) have been prepared recently in order to be used in active packaging because of their controllable and long-life antioxidant activity [2,3,4,5,6,7,8,9].

Essential oils are volatile, natural liquids with oily texture that can be extracted from several plants [10,11]. Due to the aroma character of EOs, they have been widely used in cosmetic industry [10]. In addition, the large bioactivity of EOs has being confirmed by several studies and includes antibacterial, anti-inflammatory, antifungal, antimutagenic, antineoplasmatic and antioxidant activities, along with other miscellaneous activities [12]. Oregano (*Origanum vulgare L*.) [13], thyme (*Thymus vulgaris L*.) and basil (*Ocimum basilicum*) are aromatic plants with a wide distribution throughout the Mediterranean area and especially Greece [14]. The oregano essential oils comprise more than 20 ingredients, most of which are phenolic antioxidants [15] having antimicrobial [16] properties. The primarily components of oregano essential oil are carvacrol and thymol ranging to over 80 wt %, while less abundant compounds include p-cymene, γ-terpinene, caryophyllene, spathulenol, germacrene-D, β-fenchyl alcohol and δ-terpineol [17]. Thyme oil, the essential oil of common thyme, containing mainly the phenols thymol and carvacrol [18], while in small quantities p-cymene, myrcene, borneol, and linalool [19]. Most of them show antioxidant and antimicrobial properties against a broad spectrum of gram-negative or gram-positive bacteria. Similarly, basil essential oil consists of more than 30 compounds with the main constituent being estragole [20,21]. Basil EO has been proved to exhibit antimicrobial effect against different bacteria [22] and against different fungi [23].

In order to incorporate additives, such as antioxidants, in a polymer matrix usually the melt mixing technique in screw extruders is used in industry. However, use of volatile materials, for example essential oils, in such processes is restricted due to their possible rapid loss via evaporation [24,25,26]. A controlled release of EO into the polymers would be required to overcome this rapid decline of activity. Therefore, it was anticipated that the required controlled release and protection against polymer processing conditions could be provided when the EOs are adsorbed onto an inorganic porous material [7,27]. Nano-montmorillonite (MMT) has been proposed as an ideal adsorbent and nano-carrier of EOs due to its high cation exchange capacity, high surface area and ability to swell [28,29,30]. Accordingly, several essential oils, including those from oregano (OO), thyme (TO) and basil (BO) were encapsulated in sodium montmorillonite (NaMMT) and commercially available organo-modified montmorillonite (orgMMT). The prepared NaMMTEO and orgMMTEO nanohybrids exhibited controllable and sustained antioxidant activity. Furthermore, they were melt-blended with low-density polyethylene (LDPE) to form packaging films with potential antioxidant properties. Indeed, these films retained up to 50–70% of their antioxidant activity after six months of incubation [8].

Synthesis of polymer-based nano-composites has been the subject of many investigations recently and various techniques have been developed [31,32,33,34]. Three of them are the most studied, namely, melt blending, solution mixing and in-situ polymerization. Melt-blending is the most industrially used technique since it does not require any additional equipment and can be integrated in any plastics producing factory. However, temperatures above the melting point of the polymer are needed, while a homogeneous distribution of nanometer-scale particles in highly viscous polymer melts is difficultly achieved and particle aggregation cannot be avoided. The second technique is rather simple and extensively used (mainly in laboratory experiments) though usually large amounts of potentially toxic solvents are needed. In the in situ polymerization, a good dispersion of the usually-modified layered silicate in the liquid monomer is initially achieved, resulting in homogeneous dispersion of the nano-additive in the polymer matrix. This makes it an attractive alternative route not requiring high temperatures to melt the polymer. The monomer migrates into the layered silicate galleries and polymerization often occurs between the intercalated sheets. When both intra- and extra-gallery polymerization takes place, the clay layers are delaminated and exfoliated nanocomposites can be formed. The in situ polymerization in the presence of several nano-additives was the subject of extensive experimental work by our group [35,36,37,38,39]. The main concern with this technique has to do with the fact that some specific functional groups present on the nanofiller surface could react with the radicals generated during polymerization. In fact, it has been found that primary initiator radicals may react with hydroxyl groups present on the surface of graphene oxide resulting in lower polymerization rates [39,40]. In addition, the presence of layered silica inside the reacting mixture may affect diffusion-controlled phenomena taking place during the reaction [35].

Polystyrene (PS) is a high transparent, hard, brittle and low strength polymer, used for the manufacturing of disposable containers in food packaging, where it comes in two forms, rigid and foam. Molded polystyrene used to produce the rigid form find applications as protective packaging in such products as clear food containers for dairy products, salad bars, etc., plates, bowls, disposable beverage cups and their lids, egg cartons, food trays, straws, etc. Because, of its low gas barrier, PS can be used when a breathable film is required. Expanded polystyrene in foam form (usually known by its trade name styrofoam^TM^) is used for take-out foods, plates, insulated beverage cups and bowls, hinged containers (“clamshells”) and cafeteria trays. Foods packaged in PS include yogurt, ice cream, coffee, fruit juices, and meat and fresh products. It can be recognized it by its recycling code of 6.

In order to examine if the in situ polymerization could be used as an effective technique to prepare nanocomposite materials based on sodium montmorillonite NaMMTEO or organo-modified montmorillonite (orgMMTEO) nanohybrids the bulk radical polymerization kinetics of polystyrene was investigated here. Thus, NaMMTEO and orgMMTEO nanostructures with medium (60 wt %), and high (80 wt %) EO nominal content were used as additives to styrene polymerized at a specific temperature using benzoyl peroxide as initiator. Polymerization kinetics was investigated gravimetrically by recording the variation of monomer conversion with time. The PS/NaMMTEO and PS/orgMMTEO nanohybrids were characterized using a variety of experimental techniques including, X-ray diffraction (XRD), FTIR spectroscopy, gel permeation chromatography (GPC), differential scanning calorimetry (DSC) and thermogravimetric analysis (TGA). In this way it was challenging to check how surface functional groups of the EOs could affect the kinetics ant the properties of the prepared polymer nanocomposites. Furthermore, in order to discriminate between the effect of the MMT itself or the characteristic compounds of the essential oils on the reaction kinetics and polymer properties, additional experiments carried out using styrene and model thymol, carvacrol and estragol this time. It should be emphasized here that, according to our knowledge, this is the first study on the polymerization kinetics of styrene in the presence of nano-montmorillonite including essential oils of thyme, oregano and basil.

## 2. Materials and Methods

### 2.1. Materials

The monomer used, i.e., styrene (S) with a purity ≥ 99% was purchased from Merck Schuchardt, Darmstadt, germany (Index-No: 601-026-00-0) and the hydroquinone inhibitor was removed by passing it, at least twice, through disposable inhibitor-remover packed columns, supplied from Aldrich, Darmstadt, Germany. The free radical initiator, benzoyl peroxide (BPO) with a purity > 97% (CAS: 94-36-0) was provided by Alfa Aesar, Kandel, Germany and purified by fractional recrystallization twice from methanol (from Riedel-de Haen, Munich, Germany). Dichloromethane and methanol used in the dissolution and re-precipitation of the polymer were purchased from Alfa Aesar, Kandel, Germany.

**Scheme 1:**
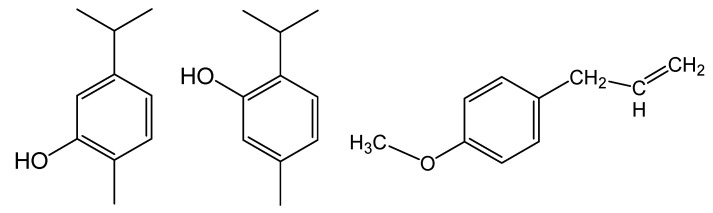
Chemical structure of thymol, carvacrol and estragol.

Origanum (OO), thyme (TO) and basil (BO) essential oils were purchased from Esperis spa., Greece according to safety data sheets, the % mass composition of oregano oil was 60–70% carvacrol, 10–12.5% thymol, 10–12.5% paracymene, 5–7% alpha-pinene, 5–7% 1-Isopropyl-4-methyl-1,4-cyclohexadiene p-Mentha-1,4-diene and 1–3% terpinen-4-olo, beta-myrcene and (R)-p-mentha-1,8-diene. Thyme oil consisted of 50–60% thymol, 15–20% para-cymene, 10–12.5% 1-Isopropyl-4-methyl-1,4-cyclohexadiene p-Mentha-1,4-diene, 3–5% carvacrol and 1–3% linalool, beta caryophyllene, beta-myrcene, (R)-p-mentha-1,8-diene, alpha-pinene, borneol and terpinen-4-olo. Basil oil consisted of 70–80% estragol, 7.5–10% linalool, 1–3% eukalyptol, 0.5–1.0% eugenol and 0.5–1.0% D-limonene. In addition, model compounds thymol (Sigma-Aldrich, Darmstadt, Germany, purity >99%), Carvacrol (Sigma-Aldrich, Darmstadt, Germany, purity >99%) and estragol (Siga-Aldrih, Darmstadt, Germany, purity >97%) were used. The chemical structure of the main components of the three EO used, i.e., thymol, carvacrol and estragol appear in Scheme 1.

Two types of clay were used: (i) sodium exchanged montmorillonite (NaMMT) with code name Nanomer^®^ PGV with mass density 2.6 g/cm^3^ and CEC value 145 meq/100 g produced by Nanocor Inc. 2870 Forbs Avenue Hoffman Estates, IL, USA and supplied by Sigma-Aldrich, Darmstadt, Germany. The chemical composition of NaMMT was 62.9% SiO_2_, 19.6% Al_2_O_3_, 3.35% Fe_2_O_3_, 3.05% MgO, 1.68% CaO, 1.53% Na_2_O. (ii) organo-modified montmorillonite (orgMMT) NANOMER^®^-I·44P produced by Nanocor Inc. 2870 Forbs Avenue Hoffman Estates, IL USA and supplied by Sigma-Aldrich, Darmstadt, Germany. NANOMER^®^-I.44P is an -onium ion modified clay containing ~40 wt % dimethyl dialkyl (C14-18) ammonium organic modifier.

All other chemicals used were of analytical grade and were used as received without further purification.

### 2.2. Preparation of NaMMT/EO and orgMMT/EO Hybrids

Loading of EO into NaMMT and orgMMT was carried out via an adsorption/evaporation procedure without the addition of organic solvents [7]. Before the adsorption/evaporation procedure, NaMMT and orgMMT were dried for 24 h in 120 °C, and then 5 g of each clay was spread in an aluminum beaker. In the middle of the aluminum beaker a smaller quartz beaker was placed and was filled with appropriate quantity of each EO. The amount of EO used was 3.0, or 4.0 g to achieve final nominal composition of EO to clays 60 and 80 wt % respectively. Then, the aluminum beaker was sealed and put in an oven at 120 °C for 24 h. Under these conditions the most volatile EO components were evaporated and adsorbed into NaMMT and orgMMT (nonvolatile fraction was ~30–60%). When the evaporation–adsorption procedure ended (after 24 h) the obtained NaMMT/EO and OrgMMT/EO clay hybrids were labeled and put in sealed closed glass beakers for further characterization.

### 2.3. Preparation of the Initial Monomer/MMT Mixtures

The monomer (S) with the sodium MMT or the organomodified MMT, were positioned to ultrasonication (in Transsonic 460H ultrasonic bath from Elma) for one hour, so that to have a satisfactory colloidal dispersion of the clay to the solution. In the final suspension, the initiator, BPO 0.03 M was added and the mixture degassed by passing nitrogen and immediately used.

### 2.4. Synthesis of PS/MMTEO Nanocomposites by the In-Situ Bulk Radical Polymerization Technique

For the preparation of the nanocomposite materials both sodium (NaMMT) and organomodified montmorillonite (orgMMT) were used at a percentage 3.0 wt % relative to monomer. The amount of montmorillonite added was kept in a relatively low percentage, since according to literature nano-MMT can significantly improve the properties of a nanocomposite material even at very low loadings [40]. Three essential oils were loaded to MMT, from thyme, oregano and basil at two nominal composition of EO to clay 60 and 80 wt % respectively. Code names and the details of the chemical conditions that were used for the preparation of all NaMt/EO and OrgMt/EO clay hybrids were included in Table 1. All clays, before polymerization were placed in an oven for 1 h at 80 °C for the removal of water possibly adsorbed in their surface. Afterwards, they were stored in desiccators until use.

In order to study the reaction kinetics, the in situ, bulk, free radical polymerization technique was employed. Polymerization was carried out in small test-tubes by heating the initial monomer-clay-initiator mixture at 80 °C for a suitable time. Polymerization temperature was kept constant in all experiments. According to this technique, 1 mL of the pre-weighed mixture of monomer with the initiator and each amount of clay were placed into a series of 10 small test-tubes. After degassing with nitrogen, they were sealed and placed into a pre-heated bath at the desired polymerization temperature. Each test-tube was removed from the bath at the pre-specified time intervals and was immediately frozen, after the addition of a few drops of hydroquinone, in order to stop the reaction. The product was isolated after dissolution in CH_2_Cl_2_ and re-precipitation in MeOH. Subsequently, all isolated materials were dried to constant weight in a vacuum oven at room temperature. All final samples were weighed and the degree of conversion was estimated gravimetrically. Duplicate experiments were run in order to check the reproducibility of the results.

Neat polymer (PS) was also synthesized under the same experimental conditions and used as reference material.

### 2.5. Synthesis of Composites Based on PS with Model Thymol, Carvacrol and Estragol

In order to check the effect of the main constituents of the essential oils, i.e., thymol, carvacrol and estragol alone on the polymerization kinetics of styrene, additional experiments were carried out using exactly the same experimental conditions (i.e., temperature, initiator, polymerization technique, etc.). Instead of the clay nanohybrids, different amounts of the three compounds were used each time. The amount of thymol, carvacrol and estragol added were 0.1, 0.5 and 1.0 wt % based on the monomer styrene. Relatively low percentages were used in order to be in the same order of magnitude with previous experiments. The materials produced were given the code names PS/Thym0.1, PS/Thym0.5, PS/Thym1.0, PS/Carv0.1, PS/Carv0.5, PS/Carv1.0, PS/Estr0.1, PS/Estr0.5 and PS/ESTR1.0 depending on the compound added and its percentage.

### 2.6. Measurements

*X-ray diffraction*. X-ray diffraction (XRD) was used to characterize the crystalline structure of MMT and the prepared PS/MMTEO materials. The instrument used was a Rigaku Miniflex II, (Chalgrove, Oxford, UK) equipped with CuKa generator (*λ* = 0.1540 nm). The XRD patterns were recorded at the range 2*θ* = 2–30° with scan speed of 2° min^−1^.

*Fourier-transform infra-red (FTIR) spectroscopy.* The chemical structure of neat PS and its nanocomposites with the clay and the clay/essential oil hybrid materials was confirmed by recording their IR spectra. The instrument used was the Spectrum 1 spectrophotometer (Perkin Elmer, Waltham, MA, USA) with an attenuated total reflectance (ATR) device. Spectra were recorded over the range from 4000 to 650 cm^−1^ at a resolution of 2 cm^−1^ and 32 scans were averaged to reduce noise. The instrument’s software was used to identify the peaks.

*Gel permeation chromatography (GPC).* GPC was used to determine the molecular weight distribution (MWD) and the average molecular weights of neat PS and all the nanocomposites of PS with NaMMTEO and orgMMTEO. The instrument used was the PL-GPC 50 Plus from Polymer Laboratories (Church Stretton, UK). It includes an isocratic pump, three PLgel 5 *μ* MIXED-C columns together with a guard pre-column in series and a differential refractive index detector. The elution solvent was tetrahydrofuran (THF) at a constant flow rate of 1 mL min^−1^. All samples were dissolved in this solvent at a constant concentration of 1 mg mL^−1^. After filtration, 200 μL of each sample was injected into the chromatograph. The column oven was kept at a constant temperature of 30 °C. Standard polystyrene samples were used for the calibration of GPC (obtained from Polymer Laboratories).

*Thermogravimetric analysis (TGA).* The thermal stability of the samples was measured by recording the mass loss with increasing temperature via thermogravimetric analysis. The instrument used was Pyris 1 TGA (from Perkin-Elmer, Waltham, MA, USA). Samples of about 8–10 mg were used and they were heated from ambient temperature to 600 °C at a heating rate of 20 °C min^−1^ under nitrogen flow.

*Differential scanning calorimetry (DSC).* DSC was used to estimate the glass transition temperature of each material prepared. The instrument used was the DSC-Diamond (from Perkin-Elmer, Waltham, MA, USA). Approximately 5–6 mg of each sample were weighed, put into the standard Perkin-Elmer sample pan, sealed and placed into the appropriate position of the instrument. Subsequently, a cycle heating-cooling-re-heating was used. The temperature program used included: heating from ambient temperature to 180 °C at a rate of 10 °C min^−1^ to ensure complete polymerization of the residual monomer, cooling from 180 to 30 °C at the same rate and heating again from 30 to 130 °C at a rate of 20 °C min^−1^. The glass transition temperature was measured from the second heating, using the half Cp interpolation method.

## 3. Results

### 3.1. Characterization of the PS/MMTEO Nanocomposites

**Figure 1:**
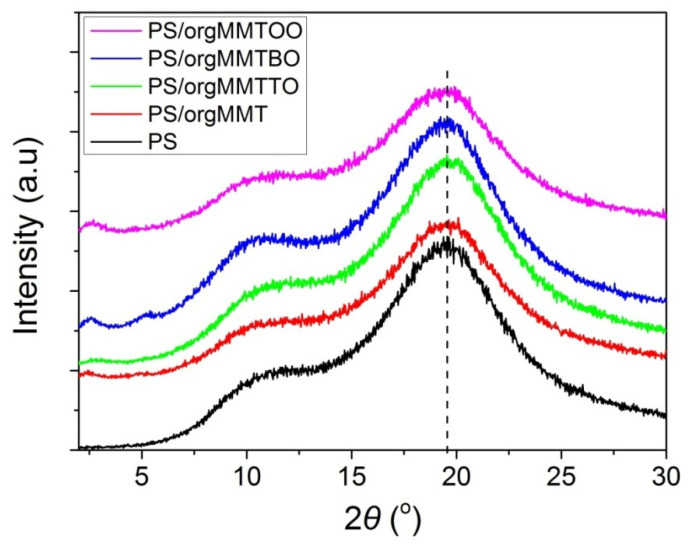
X-ray diffraction patterns of neat polystyrene (PS) and its nanocomposites with 3% orgMMT, orgMMTBO, orgMMTTO and orgMMTOO over the full range of 2θ from 2 to 30°.

Polymer–clay nanocomposites can be classified as immiscible (tactoids), intercalated, partially exfoliated, or exfoliated, depending on the clay content, the chemical nature of the organic modifier, and the synthetic method [35]. In order to identify possible exfoliation or intercalation of the clays due to the presence of the essential oils, XRD measurements were carried for neat PS, the orgMMT and the nanocomposites of PS/orgMMT, PS/orgMMTEO, PS/NaMMT and PS/NaMMTEO. XRD patterns were recorded in the angle range of 2*θ* from 2° to 30° and indicative spectra are shown in Figure 1. The amorphous structure of polystyrene was identified by the presence of a very broad peak at 19.5°. When orgMMT or NaMMT with the EΟs was incorporated into the polymer matrix, similar spectra were recorded for all nanocomposites in the range from 5 to 30°, meaning that all materials produced still present an amorphous structure.

**Figure 2:**
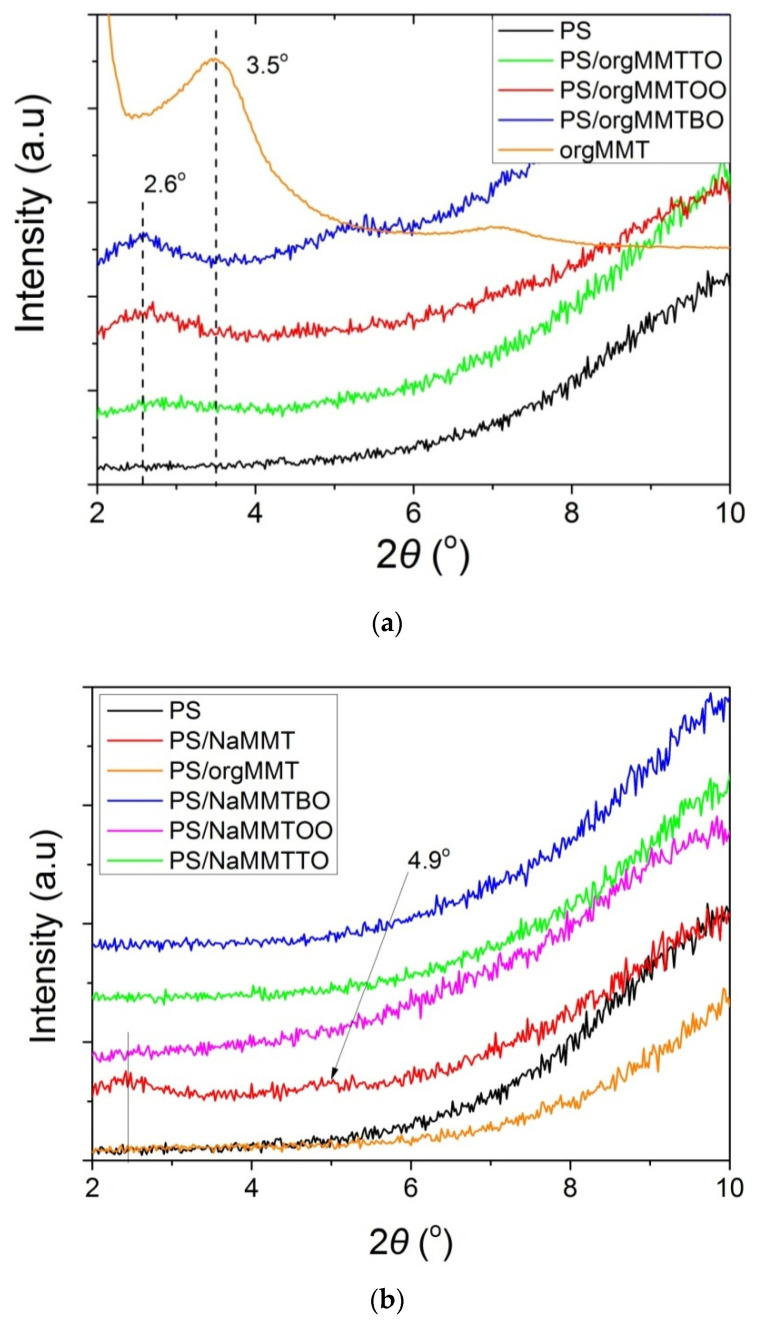
XRD patterns of organomodified MMT (orgMMT), neat PS and PS with 3% orgMMTEO nanocomposites (**a**) and of PS with NaMMTEO nanocomposites (**b**) focusing on the 2θ range from 2 to 10°.

Furthermore, we focused on small angles to investigate the morphology of the clay in the nanocomposite. The results are illustrated in Figure 2a,b. The orgMMT shows a sharp peak at 3.5° (Figure 2a) corresponding to d-spacing of 2.52 nm. The value measured in our previous publication was 2.8 nm [7]. Furthermore, the 001 reflections (Figure 2a) of all PS/orgMMTEO nanocomposites showed a rather small peak near 2.6° (ranging from 2.6 to 2.64°) and the calculated d_001_-spacing value using Bragg’s law was 3.4 nm (ranging from 3.34 to 3.4 nm). Therefore, an increase of the basal spacing in orgMMT was observed due to the insertion of the EO and further polymerization. This is an indication that intercalated structures were obtained when initially the EOs were inserted between the clay layers and furthermore due to polymerization from the insertion of macromolecular chains. An increase of d-spacing from 2.8 to 3.6 nm was observed in neat organomodified clay when the hybrid materials with the specific essential oils were produced [7]. Similar spectra were recorded for all EOs. Additionally, when organomodified clay was added to PMMA during in situ polymerization an increase of d_001_-spacing from 2.9 to 3.5 nm was measured [35]. Therefore, the values measured here are similar to those previously reported for similar systems and it is suggested that the nanocomposites can be considered as partially intercalated. For the PS/NaMMT material small peaks appeared at 2.5 and 4.6°, whereas for all the PS/NaMMTEO based nanocomposites no clear peaks were observed (Figure 2b) meaning that a rather exfoliated structure was obtained.

**Figure 3:**
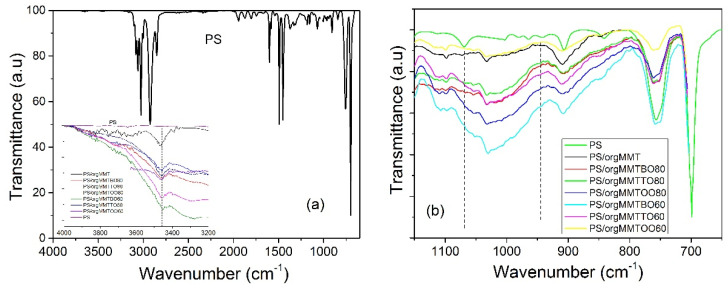
FTIR spectra of neat PS (**a**) and PS/orgMMTEO nanocomposites in the wavenumber range 4000–3200 cm^−1^ (**a**) and 1150–650 cm^−1^ (**b**).

Furthermore, in order to characterize the materials formed and confirm various functional groups, the FTIR spectra of all nanocomposites were measured and results compared to pristine PS. Indicative curves appear in Figure 3. For pristine PS (Figure 3a) the characteristic peaks observed at 3082, 3060 and 3026 cm^−1^ are assigned as aromatic stretching vibrations, *ν*(C–H)_ar_. The characteristic absorption bands at 2924 and 2850 cm^−1^ are assigned to aliphatic C–H stretching vibrations, *ν*(C–H). The other peaks at 1493 and 1452 cm^−1^ to C=C stretching vibrations, *ν*(C–C). Mono substituted benzene are obtained at *δ*(C–H)_ar_ 756 cm^−1^ and *γ*_ring_ 698 cm^−1^. All these values are in accordance to corresponding literature data [41,42].

In the embedded figure in Figure 3a a broad peak at wavenumber 3457 cm^−1^ appears in all nanocomposites, not present in neat PS, which is designated as the O–H stretching modes of interlayer H_2_O molecules. Compared to pristine PS the nanocomposites show a new broad band ranging from 1069 to 944 cm^−1^ indicating Si–O stretching (Figure 3b). It should be pointed here that peaks were very weak due to the low amount of the clay inside the polymer matrix (3%). From these indicative peaks, the presence of organoclay in the PS polymer is confirmed. 

### 3.2. Polymerization Kinetics

Polymerization kinetics was followed by recording the variation of monomer conversion with time. In order to investigate the effect of the clays with the essential oils on the polymerization of styrene, a brief review of the reaction mechanism and kinetics is presented next.

As it is well-known, polymerization of styrene follows the typical radical polymerization mechanism including the following elementary reactions:

*Initiation* (thermal decomposition of the initiator, I, to form two benzoyloxy primary radicals, R*) 
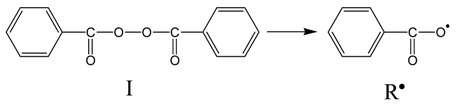


Further, the benzoyloxy radicals react with the double bond of a monomer molecule to initiate polymerization: 
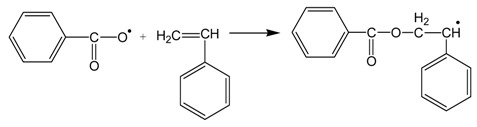


*Propagation* (radicals react with monomer molecules to form long radical chains): 
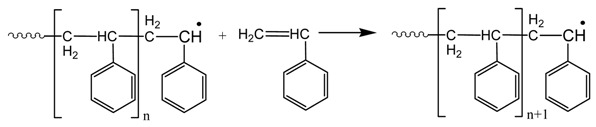


*Termination* (two macroradicals react to form a dead polymer molecule) 
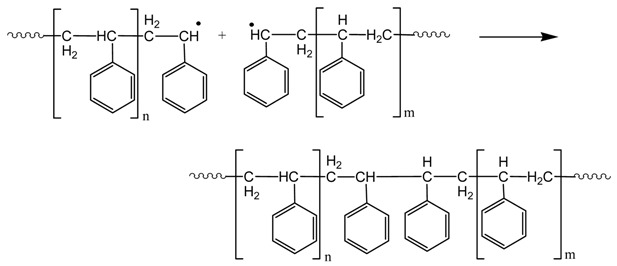


It should be noted that termination may take place by combination (as it is shown in the reaction above) or by disproportionation with transfer of a β-Hydrogen and formation of a terminal double bond. However, in polystyrene, termination by combination is the dominant mechanism of termination. Furthermore, chain transfer to monomer reactions may take place, though they are not so important during polymerization of styrene.

Based on the above reaction mechanism, the polymerization rate, denoted by the variation of monomer conversion, *X* with time, *t* can be expressed by the following equation, [43].

(1) $$\frac{dX}{dt}=\left(k_{p}+k_{trM}\right){\left(\frac{fk_{d}[I]}{k_{t}}\right)}^{1/2}(1-X)$$

where *k_d_, k_p_, k_trM_* and *k_t_* denote the kinetic rate coefficients of the initiator decomposition, propagation, chain transfer to monomer and termination reactions, *f* is the initiator’s efficiency. Note that in Equation (1) for simplicity reasons the steady-state approximation for the initiator primary radicals and the total radical population has been applied, which have been proven to hold at low monomer conversions. The initiator concentration, [I], varies with time according to:

(2) $$\frac{d[I]}{dt}=-k_{d}[I]\Rightarrow [I]={\left[I\right]}_{0}\exp\left(-k_{d}t\right)$$

In order to integrate Equation (1) and provide an expression for the variation of monomer conversion with time, all kinetic rate coefficients and *f* are assumed independent of conversion. This is correct at low monomer conversions, where diffusion-controlled phenomena do not affect the polymerization kinetics. Then, by replacing Equations (2) to (1) and integrating, one gets:

(3) $$X=1-\exp\left[2k_{eff}\left(1-e^{-k_{d}t/2}\right)\right]$$

with

(4) $$k_{eff}=\left(k_{p}+k_{trM}\right){\left(\frac{f{\left[I\right]}_{0}}{k_{d}k_{t}}\right)}^{1/2}$$

The kinetic rate constants for the polymerization of styrene can be evaluated from the following Arrhenius expressions, denoting variation only with the reaction temperature, T: *k_p_* = 4.27 × 10^7^ exp(−32,500/RT) L/mol/s, *k_t_* = 1.255 × 10^6^ exp(−846/T) L/mol/s, *k_d_* = 5 × 10^16^ exp(−143,000/RT) s^−1^, *k_trM_* = 5 × 10^−5^ *k_p_* and *f* = 1 [44]. All experiments in this study were performed at 80 °C. At this temperature, the values of the above kinetic rate constants, are: *k_p_* = 664.5 L/mol/s, *k_t_* = 1.14 × 10^8^ L/mol/s, *k_d_* = 3.5 × 10^−5^ s^−1^, *k_trM_* = 0.033 L/mol/s and f = 1. Then, the variation of monomer conversion with time can be theoretically estimated from Equation (3) setting the initial initiator concentration as [*I*]_0_ = 0.03 mol/L. It should be pointed here that Equation (3) is valid only at low monomer conversions where diffusion-controlled phenomena do not affect the chemical reactions.

**Figure 4:**
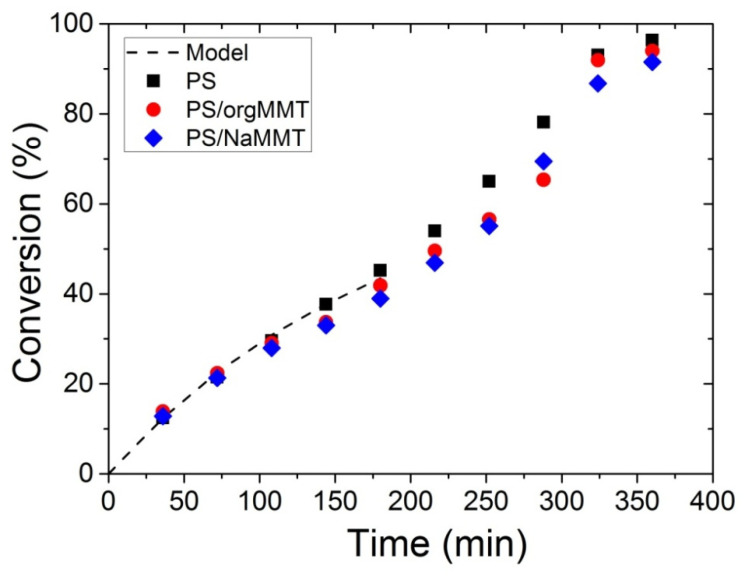
Variation of conversion with time of neat PS and PS/NaMMT or PS/orgMMT at 80 °C with initiator BPO at 0.03 M showing the effect of the nanoclays on the bulk polymerization kinetics of PS. The straight line represents result of the theoretical kinetic model.

The experimental data showing the effect of adding sodium or organomodified clay on the PS polymerization kinetics are illustrated in Figure 4. Results of the theoretical model, according to Equation (3), are also included in this figure as a continuous line. It is obvious that the theoretical model simulates very well the experimental data of neat polystyrene at low monomer conversions. This is also verification that the experimental measurements are accurate. Therefore, it seems that typical radical polymerization kinetics is followed for this monomer until almost 40% conversion. Afterwards, an increase in the conversion values and as a result of the reaction rate is observed. This is ascribed to the well-studied auto-acceleration phenomenon or gel-effect. Accordingly, when a significant amount of macromolecular chains are produced, chain entanglements hinder the movement of macroradicals to find one another and terminate. Therefore, radical bimolecular termination is reduced due to the presence of the polymer chains, resulting in a local increase of their concentration. Hence, according to equation (1), the polymerization rate is enhanced resulting in higher conversion vales at the same reaction time. At very high conversions, beyond 90%, even the movement of the small monomer molecules or the initiator primary radicals is hindered. This results in reduced propagation of monomers or reduced initiator efficiency leading to a polymerization rate tending to zero before the full consumption of the monomer. This phenomenon is more pronounced when the polymerization temperature (80 °C in this investigation) is below the glass transition temperature of the polymer (near 95 °C for PS) and is reported as glass-effect. Thus final monomer conversions are always less than 100% [44].

From Figure 4 it is clear that addition of the neat nanoclay results in a slight reduction of the polymerization rate compared to neat PS. This has been also observed in literature and is attributed to the fact that nano-clay may act as a radical scavenger resulting in slightly reduced radical concentrations and as a result to slightly lower conversion values [44,45].

**Figure 5:**
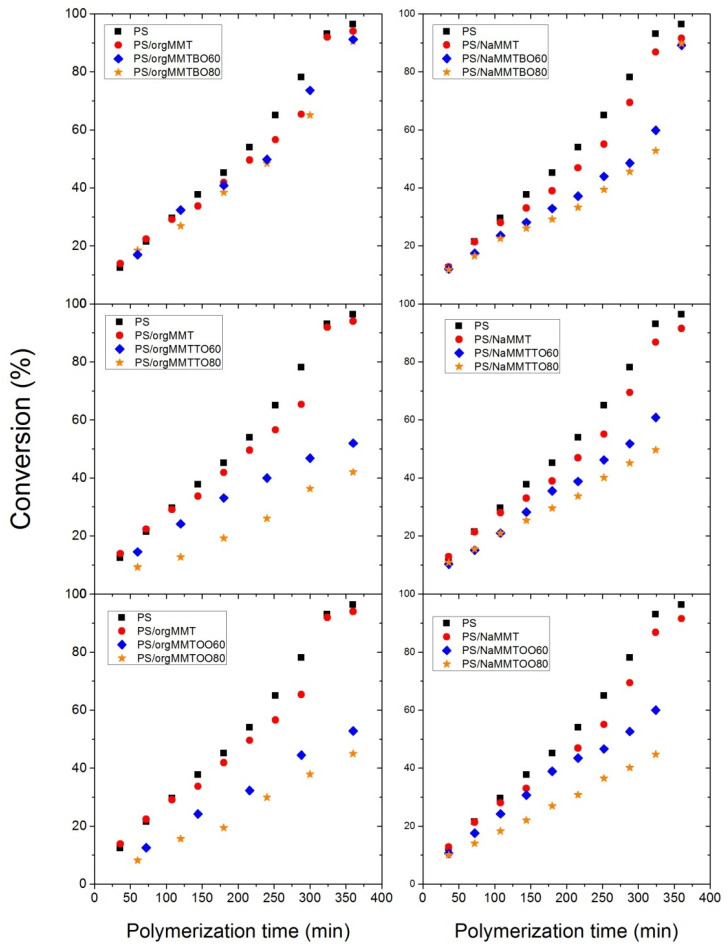
Variation of monomer conversion with time during polymerization of styrene at 80 °C with 0.03 mol/L initial BPO concentration. The left column presents results when the orgMMT with the essential oils was added, whereas the right column when the NaMMT with the EOs was added.

The effect of adding the nano-clays with the EOs on the polymerization kinetics of PS appears in Figure 5. Generally, visually it was observed that, the nano-additives based on the organomodified MMT presented a good dispersion of the nanoclays into the polymer matrix and a homogeneous suspension was obtained. In contrast, when using the sodium MMT the dispersion was not so good even after intense stirring. The variation of conversion with time was not affected much when the basil EO was used. However, a significant reduction in polymerization rate was obvious when using the oregano or thyme oil and the reduction followed the amount of the essential oil present in the clay. From the chemical composition of the three EOs used, it seems that the presence of phenols, such as thymol or carvacrol in large amounts in thyme oil and oregano oil seems to be the main reason for the reduction of the polymerization rate when using TO or OO. In contrast the main component of BO, i.e., estragol does not contain hydroxyl groups. Specifically, the presence of hydroxyl groups in these substances seems to result in side reactions with the radicals. It is known that, phenols may play a role as inhibitors in radical polymerization reactions. However, the question here is whether phenolic hydroxyls present in thymol or carvacrol react with the macro-radicals or with the primary radicals formed from the decomposition of the initiator. Moreover, do they react as chain transfer agents, inhibitors, or retarders? When a compound acts as a chain transfer agent in a radical polymerization mechanism, then it reduces the average molecular weight of the polymer but it does not affect polymerization rate since the small radicals produced can find a monomer molecule with equal reactivity as the initiator primary radicals. This is not the case here since polymerization rate was reduced. Hence the concept of chain transfer reaction is abandoned. Inhibitors are compounds that completely stop the polymerization by reacting with the radicals formed and not permitting them to find monomer molecules and start polymerization. This again was not the case here since an induction time was not observed. Then, it seems that thymol and carvacrol present in the essential oils TO and OO play the role of reaction retarder, meaning that they reduce the polymerization rate by reacting with radicals and the new radicals formed can propagate polymerization but in a lower rate.

In order to identify if the macro-radicals or the primary radicals are those that mainly react with the hydroxyl groups in retardation reactions, we performed additional measurements of the average molecular weight of the polymer formed. Accordingly, if macro-radicals take part in such reactions then they will eventually stop increasing in length through propagation resulting in polymer with lower average molecular weight. If primary initiator radicals take part in retardation reactions with the hydroxyl groups then it is the initiator efficiency that will be reduced since not all radicals produced from the decomposition of the initiator will eventually react with monomer molecules to form long chains. To clear the picture the theoretical equation providing the number average molecular weight of a polymer was considered. This can be estimated from the number average degree of polymerization, which, in the case of termination by combination, is the double of the average kinetic chain length, *ν*:

(5) $$\frac{1}{\nu }=\frac{k_{t}[P^{•}]}{k_{p}[M]}+\frac{k_{trM}[M]}{k_{p}[M]}=\frac{{\left(fk_{d}[I]k_{t}\right)}^{1/2}}{k_{p}[M]}+C_{M}$$

It should be noted here that Equation (5) provides the instantaneous average kinetic chain length which is correct at low conversion where diffusion-controlled phenomena are not present. According to Equation (5), if the initiator efficiency, *f*, is decreased then the kinetic chain length, ν and as a result the polymer number average molecular weight will be in increased. The physical picture behind this is that, if the number of primary initiator radicals is reduced and since they potentially can react with the same number of monomer molecules it will eventually result to a lower number of macro-radicals though with a higher chain length.

**Figure 6:**
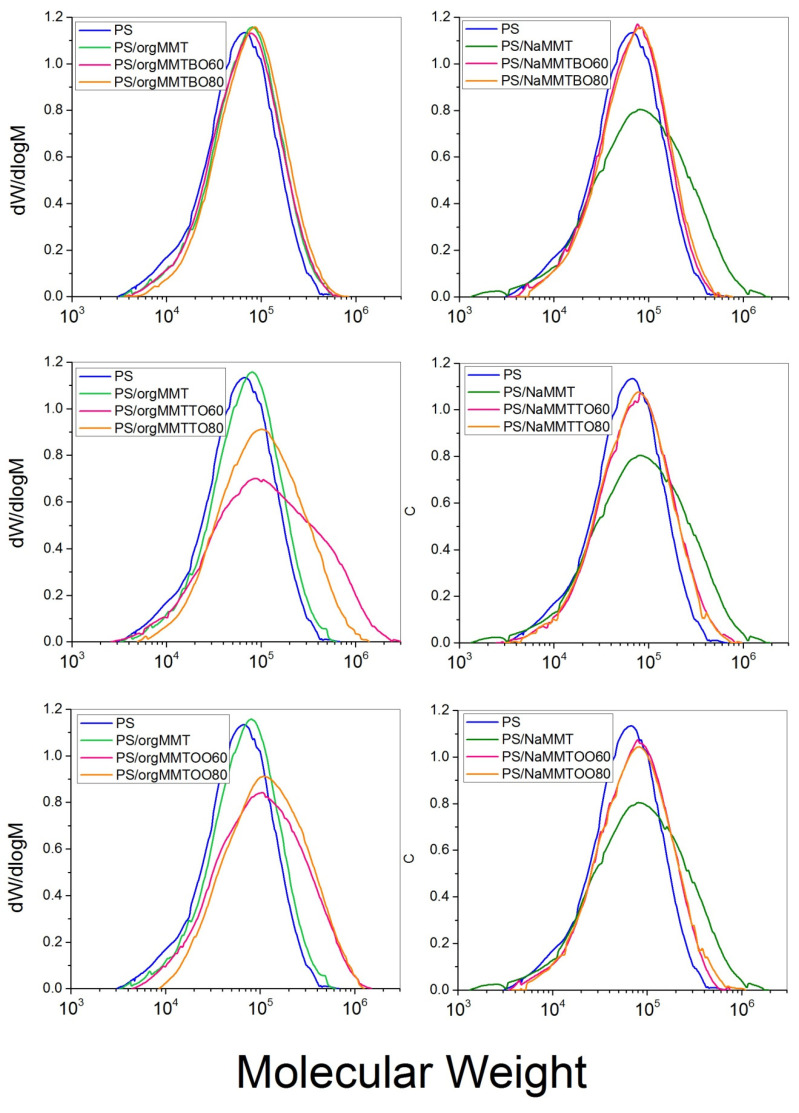
Molecular weight distribution of neat PS and its nanocomposites with either orgMMTBO, orgMMTTO, orgMMTOO (left column), or NaMMTBO, NaMMTOO and NaMMTTO (right column).

**Scheme 2:**
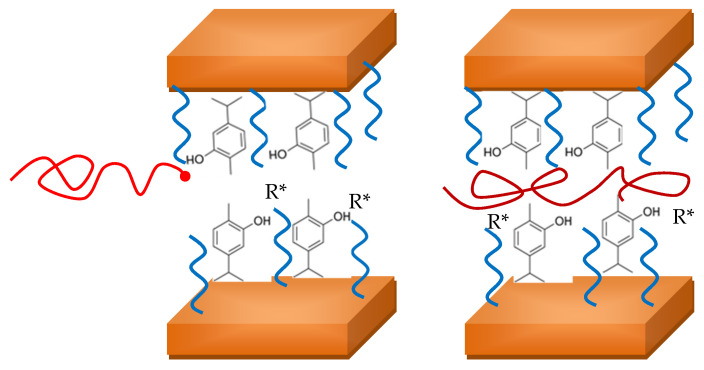
Possible chemical reactions of the primary initiator radicals, R* or the macro-radicals with the hydroxyl groups of compounds in the essential oils inside the clay galleries of the organo-modified montmorillonite.

The molecular weight distribution (MWD) and the average molecular weights of neat PS and all nano-hybrids were measured with GPC and results are presented in Figure 6 and Table 2. The number average molecular weight, Mn, of neat PS was measured at 37,120 and increased to 44,680 when orgMMT was added. This is a clear indication that orgMMT acts as a radical scavenger, in accordance with previous findings for the in situ MMA homopolymerization in the presence of organomodified montmorillonite [35,36]. Moreover, the addition of the clays with basil essential oil, orgMMTBO60 or orgMMTBO80 resulted in slightly higher values of the number average molecular weight. However, this increase was much pronounced when the essential oils of oregano or thyme were used. Actually, M_N_ increased from 44,680 to 52,200 and 61,380 when orgMMTTO60 and orgMMTTO80 were used and to 56,870 and 73,860 when orgMMTOO60 and orgMMTOO80 were used. This can be clearly seen in Figure 6, where a shift of the distribution to higher values is clear. In contrast, the MWD for PS/orgMMTBO was almost the same with that of PS/orgMMT. This is a clear indication that the presence of the hydroxyl groups in carvacrol and thymol which are placed in the galleries of the MMT can be more easily accessed by the small primary initiator radicals resulting in decreased initiator efficiency and thus to polymers with fewer macomolecular chains having higher molecular weight. A schematic representation of the phenomenon appears in Scheme 2.

**Scheme 3:**
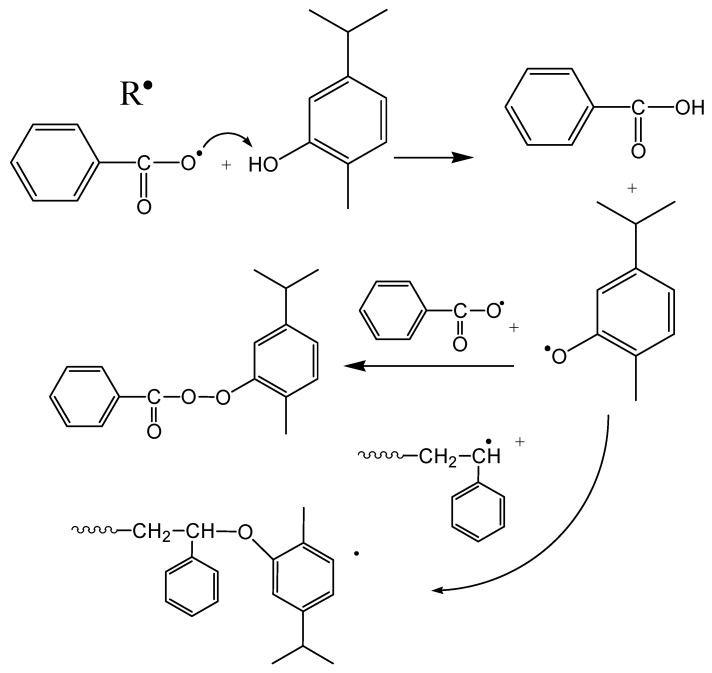
Possible reaction mechanism of the phenoxy radicals formed from the reaction of an initiator primary radicals, R* with the hydroxyl groups of thymol or carvacrol present in TO or OO either with another primary initiator radical or with a macro-radical to form inactive species.

Using chemical reactions the above observations can be explained as follows: The phenoxy radicals formed from the reaction of an initiator primary radicals, R* with the hydroxyl groups of thymol or carvacrol present in TO or OO may further react either with another primary initiator radical or with a macro-radical to form inactive species (Scheme 3). The presence of more hydroxyl groups results in lower initiator efficiency, *f* and as a result to higher average molecular weight of the polymer. This mechanism explains the lower polymerization rate together with the higher average molecular weight measured in the orgMMTOO and orgMMTTO samples.

Finally, from Figure 6 and Table 2 it is seen that using the NaMMT with the essential oils only a slight increase in the polymer average molecular weight was observed. The MWD is slightly shifted to higher values. This is because in this case mainly macro-radicals react with the hydroxyl groups and not primary radicals thus the initiator efficiency is not reduced significantly.

**Figure 7:**
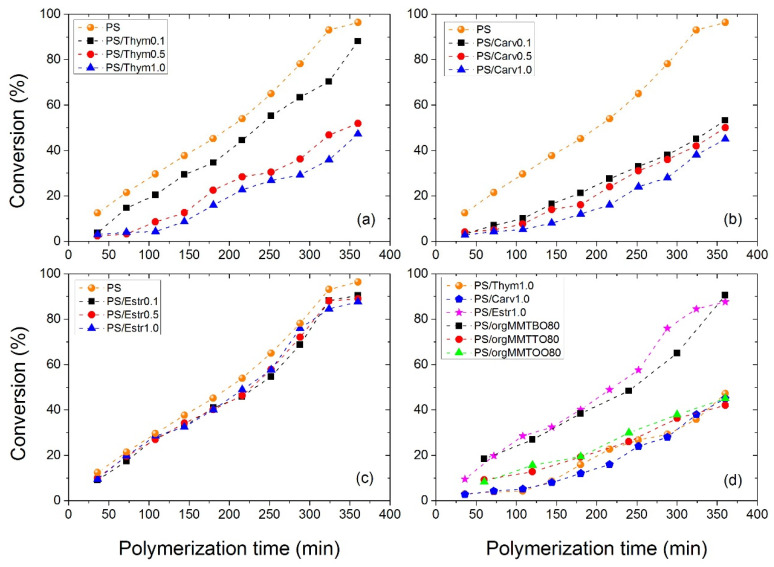
Variation of monomer conversion with time during polymerization of styrene with 0.1, 0.5 and 1.0 wt % of model thymol (a), carvacrol (b) and estragol (c) at 80 °C with 0.03 mol/L initial BPO concentration. The downright figure (d) presents comparative results of model compounds at 1.0% with orgMMTBO80, orgMMTTO80 and orgMMTOO80.

Furthermore, in order to check if the compounds of the essential oils or the clay are responsible for the specific effect on polymerization kinetics of styrene additional experiments carried out using model thymol, carvacrol and estragol at several relative to styrene percentages. Results on the variation of conversion with time appear in Figure 7. It is clear the effect of estragol on the variation of conversion with time is negligible. However, the effect of either carvacrol or thymol is very important. Specifically polymerization rate is significantly reduced and this phenomenon is more intense when the amount of the compound is increased. Therefore, these results verify the hypothesis shown in Scheme 3 that primary initiator radicals and to a lesser extend macroradicals react with the hydroxyl groups of these compounds resulting in lower radical concentration and hence lower polymerization rate. In a final attempt to reinforce these results in Figure 7d the conversion vs. time profiles obtained from the orgMMTEO with the higher amount of the EO are compared to those obtained when using 1.0% of each model compound. It is very interesting that similar results were obtained in each essential oil with the corresponding model compound. Finally, the average molecular weight of the polymer formed was measured with GPC and results are illustrated in Table 3. It is seen that in accordance with previous findings the effect of adding estragol was negligible. However, adding either thymol or carvacrol results in an increase in the average molecular weight of the polymer formed. Meaning again that, the initiator primary radicals react with the phenol hydroxyl groups resulting in lower initiator efficiency and hence lower macromolecular chains having higher degree of polymerization. The polydispersity of the MWD was not changed significantly.

### 3.3. Thermal Properties of the Materials Produced

**Figure 8:**
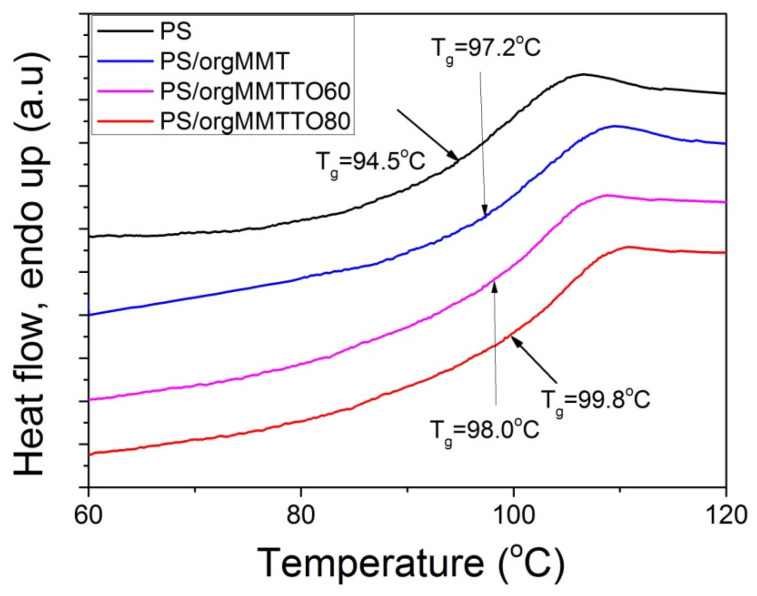
Indicative differential scanning calorimetry (DSC) data showing the estimation of the glass transition temperature, Tg of PS and some of its nanocomposites.

A “nano-effect” eminent in the literature related to the thermal properties of a polymer, has been the change in the glass transition temperature, Tg of the polymer matrix with the addition of nanosized particles. Depending upon the interaction between the matrix and the particle, both increase and decrease in the Tg have been reported [35]. In addition, the technique used for the preparation of the nanocomposite (i.e., solution casting, melt mixing or in situ polymerization) has been shown to affect Tg, with the in situ polymerization resulting usually to higher values [35,42]. Typical thermograms, obtained after the second heating, of neat PS and PS/orgMMT, PS/orgMMTTO60 and PS/orgMMTTO80 are presented in Figure 8. The values of Tg, marked in this figure and included in Table 2 are determined by the half Cp extrapolation method. In this research, the glass transition temperature of neat polystyrene was measured 94.5 °C, which is a typical value for this polymer [40,42]. The Tg value was increased to 97 °C when orgMMT was added and further increased to values between 98 and 100 °C when the organomodified clay with the essential clays was added. Moreover, the Tg with the NaMMT was slightly increased to 96 °C and when the NaMMT with the EO added Tg increased again to values varying between 97 and 100 °C. An increase of the Tg of PS from 94.2 to 96.5 °C when 3% of silica nanoparticles added was also reported in literature [42]. Furthermore, addition of 1% graphene oxide increased the Tg of PS from 90 to 96.8 °C [40]. The increase of Tg of polystyrene due to the presence of the clay is ascribed to the confinement of intercalated PS chains within the silicate galleries that prevents the segmental motions of the polymer chains.

**Figure 9:**
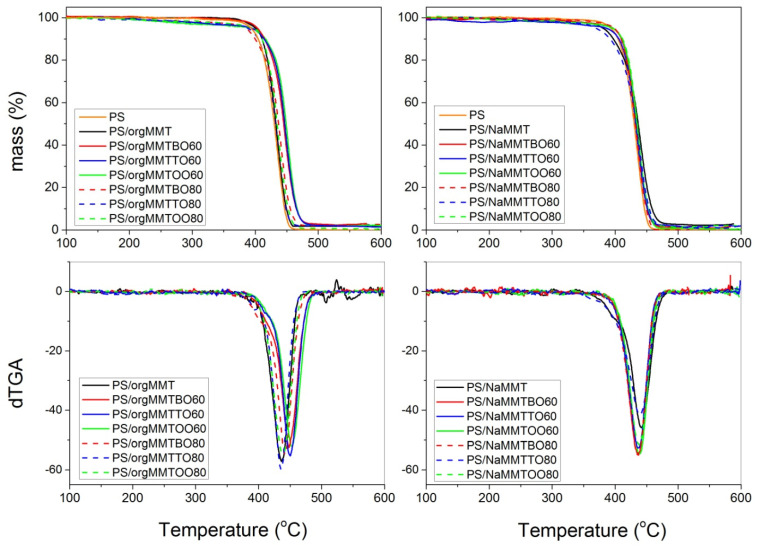
Mass loss and differential thermogravimetric analysis (TGA) curves obtained during heating at 20 °C/min of neat PS and PS/orgMMTEO and PS/NaMMTEO nanocomposites with various types of added essential oils.

Finally, thermogravimetric analysis in inert atmosphere was used to investigate the effect of the MMTEO on the thermal stability of neat PS. The variation of mass loss with temperature of both PS/orgMMTEO and PS/NaMMTEO recorded via TGA, appears in Figure 9. In the same figure differential TGA curves are also included. Specific temperatures where 4% and 20% degradation occurs, T_4%_, T_20%_ together with the temperature where degradation occurs at a maximum rate, T_max_, appear in Table 4. In general, degradation starts around 385 °C and ends at 460–470 °C leaving a residual mass at 600 °C, near zero for neat PS and around 2.0% for the MMT nanocomposites (Table 4). The reliability of the experiments was verified from literature data. As an example, the T_max_ measured here for neat PS was 435 °C which is very close to the value 437 °C measured at the same heating rate recently by Blanco et al. [46].

The thermal degradation of radically prepared polystyrene in an inert atmosphere has long been studied in literature, with most researchers reporting that the dominating mechanism is that of random scission of the carbon-carbon main chain [40,47]. In Figure 9, the differential TGA curves showed a single peak in most of the materials studied denoting thus a single-step degradation mechanism. The nano-hybrids of PS with the organomodified montmorillonite presented a slight enhancement in the thermal stability compared to neat PS. This can be seen from the T_20%_ values, where the value measured for PS, i.e., 415 °C, increased to 419 °C in the PS/orgMMT and further to 430 °C in the nanocomposites with 60 EO, whereas when the amount of the EO increased to 80 this values decreased to 416–420 °C. A similar trend was observed in the T_max_ values. Enhanced thermal properties for nanocomposites of polystyrene with organomodified clays have also been observed by other researchers [41,48]. The origin of this increase in the degradation temperatures when MMT is added in the polymer matrix has been attributed to the ability of nanometer silicate layers to obstruct volatile gas produced by thermal decomposition. Accordingly, thermal decomposition begins from the surface of the nanocomposites, leading in an increase of the orgMMT content and the formation of a ’protection layer’ by the clay. This, so-called ‘barrier model’ works well for char-forming polymers but it seems to not hold for non-char-forming polymers such as PMMA or PS [49,50]. In this case, the phenomenon can be better described using the nanoconfinements theory [49,50]. Accordingly, with the start of polymer degradation the newly formed radicals are nanoconfined, permitting a variety of bimolecular reactions to occur. As degradation progresses, a decrease in the surface free energy results in a migration of the clay platelets gradually to the surface forming the barrier that has been detected experimentally. The emission of volatile degradation products is prevented by the distributed orgMMT sheets acting as block layers. Meanwhile, the degraded small gas molecules which cannot diffuse in time would restrain the reaction of PS, leading to a higher temperature at the same mass loss. In addition, PS thermal stability can be improved from the absorption of a part of the polymer chains with low degree of degradation at the interface between polymer and MMT [51]. In the NaMMT samples the thermal degradation curves were similar to that of neat PS. A possible presence of small amounts of tactoids in the partially exfoliated structure of sodium nanocomposite could account for the not so-much increase in the decomposition rate observed in these materials.

**Figure 10:**
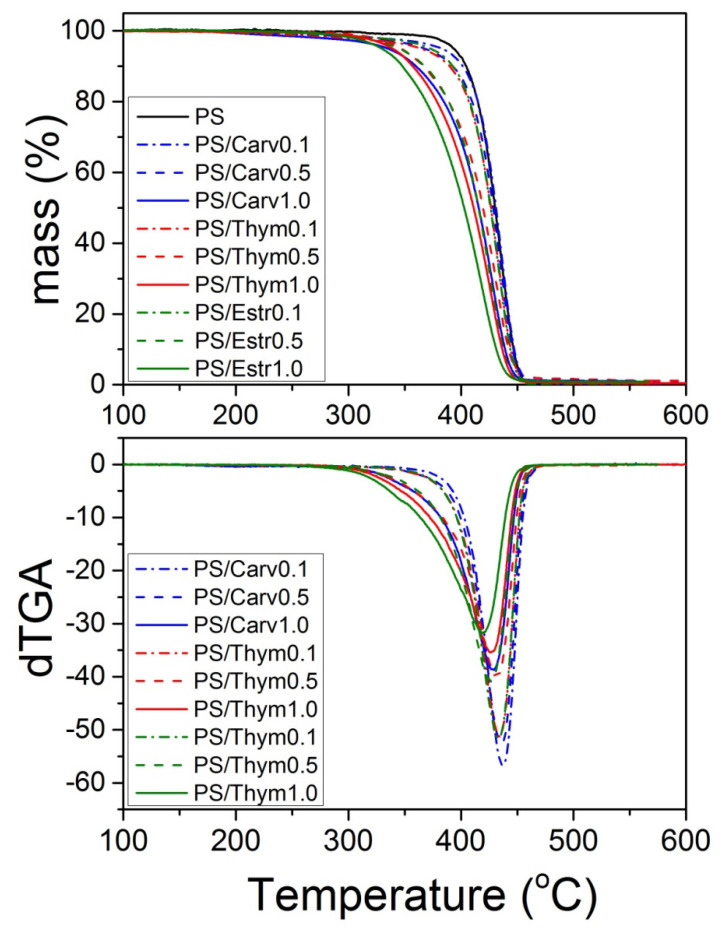
Mass loss and differential TGA curves obtained during heating at 20 °C/min of PS with model thymol, carvacrol and estragol at 0.1, 0.5 and 1.0 wt %.

In order to check if orgMMT itself or the materials with the orgMMTEO are responsible to the above findings further TGA experiments were carried out using again only the model compounds, thymol, carvacrol and estragol, which are the main constituents of the EOs studied. The results appear in Figure 10. It is clear that all the materials produced exhibit lower thermal stability compared to neat PS as can be observed form degradation staring at lower temperatures. Particularly, as the amount of thymol, carvacrol or estragol increases thermal degradation starts at much lower temperatures. Therefore, it can be concluded that using only the model compounds present in the EOs will definitely result in materials with much lower thermal degradation. It is the presence of MMT and specifically of the organomodified MMT which retains the polymer original properties or sometimes results in enhanced thermal stability.

## 4. Conclusions

This research is in the general framework of producing active plastic packaging using bio-based additives with antioxidant properties. The in situ bulk polymerization was investigated as a promising technique, not requiring high temperatures or toxic solvents, in order to incorporate additives containing possibly volatile materials into a polymer matrix. The radical polymerization of styrene was investigated in the presence of sodium montmorillonite (NaMMT) and organo-modified montmorillonite (orgMMT) including thyme (TO), oregano (OO), and basil (BO) essential oil at nominal composition 60 and 80%. Polymerization kinetics was investigated by measuring the variation of monomer conversion with time gravimetrically, as well as the molecular weight distribution and average molecular weights of the polymer formed via GPC. Better dispersion was observed when orgMMT was added instead of NaMMT. Using X-ray diffraction it was found that mainly intercalated structures are obtained when using the organically modified montmorillonite whereas mainly exfoliated when the NaMMT was employed. the incorporation of the MMTEO in the polymer matrix was verified via FTIR spectroscopy. It was found that the hydroxyl groups present in thymol and carvacrol which are the main ingredients of thyme oil and oregano oil participate in side retardation reactions leading to lower polymerization rate accompanied by higher polymer average molecular weight. This was attributed to the reactions of the primary initiator radicals with the hydroxyl groups resulting in decreased initiator efficiency. Use of basil oil, which mainly includes estragol and not active hydroxyl groups, did not seem to affect significantly the polymerization kinetics and polymer MWD. The glass transition temperature (measured from DSC) and the thermal stability (from TGA) of the nanocomposites formed were slightly increased from 95 to 98 °C and from 435 to 445 °C, respectively.

Furthermore, in order to discriminate between the effect of the MMT itself or the characteristic compounds of the essential oils on the reaction kinetics and polymer properties, additional experiments carried out using styrene and model thymol, carvacrol and estragol this time. Similar polymerization kinetics was observed when using model compounds of the chemicals included in large amounts in the essential oils with those obtained from the hybrids of orgMMTEO. In contrast, the enhancement of the thermal stability was mainly the result of the nanoclays, since using only the model compounds the thermal stability of the materials significantly decreased.

Therefore it seems that mainly the organically modified montmorillonite based materials can be used in this technique and be successfully incorporated into a polymer matrix in order to provide inherent antioxidant activity in a polymer matrix to be used in active food packaging.

## Figures and Tables

**Table 1:** Designation and amounts (wt %) of oregano oil, thyme oil, basil oil and sodium montmorillonite (NaMMT) or organo-modified montmorillonite (orgMMT) used for the preparation of all NaMMTEO hybrids and orgMMTEO hybrids.

Sample	NaMMT/orgMMT (% Relative to Monomer)	Nominal Composition of EO to Clays (%)		
		Basil	Thyme	Oregano
PS	-	-	-	-
PS/orgMMT	3	-	-	-
PS/orgMMTBO60	3	60	-	-
PS/orgMMTTO60	3	-	60	-
PS/orgMMTOO60	3	-	-	60
PS/orgMMTBO80	3	80	-	-
PS/orgMMTTO80	3	-	80	-
PS/orgMMTOO80	3	-	-	80
PS/NaMMT	3	-	-	-
PS/NaMMTBO60	3	60	-	-
PS/NaMMTTO60	3	-	60	-
PS/NaMMTOO60	3	-	-	60
PS/NaMMTBO80	3	80	-	-
PS/NaMMTTO80	3	-	80	-
PS/NaMMTOO80	3	-	-	80

**Table 2:** Number- and weight-average molecular weights (${\bar{M}}_{n}$, ${\bar{M}}_{w}$), as well as polydispersity of the MWD (PD) together with the glass transition temperature (T_g_) of neat PS and its nanocomposites with the sodium or organo-modified montmorillonite (MMT) with or without the essential oils.

Sample	${\bar{M}}_{n}$	${\bar{M}}_{w}$	PD	T_g_ (°C)
PS	37,120	77,590	2.09	94.5
PS/orgMMT	44,680	90,300	2.02	97.2
PS/orgMMTBO60	45,310	90,460	2.00	97.6
PS/orgMMTTO60	52,200	222,610	4.26	98.0
PS/orgMMTOO60	56,870	159,830	2.81	97.2
PS/orgMMTBO80	50,010	100,520	2.01	99.7
PS/orgMMTTO80	61,380	152,420	2.48	99.8
PS/orgMMTOO80	73,860	173,160	2.34	98.5
PS/NaMMT	38,610	140,120	3.63	96.0
PS/NaMMTBO60	44,070	86,450	1.96	97.1
PS/NaMMTTO60	45,750	100,440	2.20	97.4
PS/NaMMTOO60	44,960	96,390	2.14	96.8
PS/NaMMTBO80	47,510	91,830	1.93	99.3
PS/NaMMTTO80	45,900	97,260	2.12	98.9
PS/NaMMTOO80	47,810	102,900	2.15	98.2

**Table 3:** Number- and weight-average molecular weights (${\bar{M}}_{n}$, ${\bar{M}}_{w}$), as well as polydispersity of the MWD (PD) of the composite materials based on PS and thymol, carvacrol and estragol at different relative-to-monomer amounts of 0.1, 0.5 and 1.0 wt %.

Sample	${\bar{M}}_{n}$	${\bar{M}}_{w}$	PD
PS	37,120	77,590	2.09
PS/Thym0.1	38,240	81,070	2.12
PS/Thym0.5	44,880	101,430	2.26
PS/Thym1.0	54,770	154,990	2.83
PS/Carv0.1	38,270	83,420	2.18
PS/Carv0.5	46,830	101,610	2.17
PS/Carv1.0	51,930	144,360	2.78
PS/Estr0.1	37,170	80,290	2.16
PS/Estr0.5	36,800	79,500	2.16
PS/Estr1.0	38,390	81,400	2.12

**Table 4:** TGA data of neat PS and its nanocomposites with the sodium and orgno-modified MMT with or without the essential oils. Temperatures at 4% and 20% degradation, as well as at the maximum degradation rate, T_4%_, T_20%_ and T_max_ and residual mass at 600 °C.

Sample	T_4%_ (°C)	T_20%_ (°C)	T_max_ (°C)	Residual at 600 °C
PS	390	415	435	0.0
PS/orgMMT	397	419	437	1.8
PS/orgMMTBO60	400	428	448	2.3
PS/orgMMTTO60	383	430	450	2.0
PS/orgMMTOO60	387	432	452	2.2
PS/orgMMTBO80	379	417	442	2.1
PS/orgMMTTO80	380	416	437	2.0
PS/orgMMTOO80	370	420	438	1.8
PS/NaMMT	390	415	441	2.3
PS/NaMMTBO60	393	419	437	1.8
PS/NaMMTTO60	375	417	437	2.0
PS/NaMMTOO60	388	420	438	2.0
PS/NaMMTBO80	390	418	437	1.5
PS/NaMMTTO80	365	412	439	1.6
PS/NaMMTOO80	388	421	437	1.7
